# Leaf- and root-associated bacterial communities differ in their resistance and resilience to N disturbance in a temperate steppe

**DOI:** 10.1128/aem.00332-26

**Published:** 2026-05-27

**Authors:** Chunyan Guo, An Yang, Xueqing Zhang, Wenming Bai, Wen-Hao Zhang

**Affiliations:** 1 Key Laboratory of Vegetation and Environmental Change, Institute of Botany, Chinese Academy of Sciences, Beijing, China; 2 College of Resource and Environment, University of Chinese Academy of Sciences 74519, Beijing, China; The University of Tennessee Knoxville, Knoxville, Tennessee, USA

**Keywords:** leaf- and root-associated bacterial communities, N addition and cessation of N addition, plant functional traits, resistance and resilience, temperate grasslands

## Abstract

Enhanced deposition of nitrogen (N) has great impacts on grassland ecosystems. A decline in N deposition has occurred in many regions across the globe. Changes in N deposition alter the structure and functions of grassland ecosystems and bacterial community of soil and rhizosphere. However, the responses of plant microbiomes to N deposition and cessation of N input in terms of resistance and resilience have not been systematically evaluated. We examined the effects of N addition and cessation of N addition on leaf- and root-associated bacterial communities through a consecutive N addition and cessation of N addition experiment in a temperate grassland. We found that leaf-associated bacterial community exhibited lower resistance to N enrichment than root-associated bacterial community, which was mainly steered by leaf soluble sugars and leaf morphology *via* regulating functional taxa. In contrast, the root-associated bacterial community showed stronger resilience to cessation of N addition than leaf-associated bacterial community, which may be explained by the high N accumulation in roots and root morphology *via* regulating functional taxa. The greater resistance and resilience in the root-associated bacterial community may be attributed to the presence of host-related factors. Additionally, N enrichment-induced suppression of beneficial symbiotic microbes associated with the N cycle in the leaf-associated bacterial community was not readily recovered after cessation of N input. Conversely, microbes involved in carbon cycle and ecological restoration in the root-associated bacterial community showed a quick recovery after cessation of N enrichment. Our results offer valuable insights into the mechanisms by which changes in N input influence the plant microbial community.

**IMPORTANCE:**

As an integral component of ecosystems, the plant microbiome plays an important role in the response of grassland ecosystems to enhanced N deposition. Changes in N deposition influence bacterial communities of soil and rhizosphere of grassland ecosystems. However, whether and how the N deposition and cessation of N input impact microbiomes of plant species of temperate grasslands remain unexplored. Based on a long-term N-addition experiment in a temperate steppe, we discover that leaf- and root-associated bacterial communities respond differently to N addition and subsequent cessation of N addition. The leaf-associated bacterial community exhibits lower resistance to N enrichment than the root-associated bacterial community due to the unique environment of the phyllosphere, whereas the root-associated bacterial community shows stronger resilience to cessation of N addition than the leaf-associated bacterial community due mainly to the higher root N accumulation and morphology. These findings offer valuable insights into the impact and mechanism of N interference on the plant microbial community.

## INTRODUCTION

Enhanced atmospheric nitrogen (N) deposition due to anthropogenic activities has great impacts on stability (1, 2) and functioning of terrestrial ecosystems (3–7). A decline in atmospheric N deposition has occurred in many regions across the globe due to the implementation of emission policies in recent decades (8, 9). Studies on the recovery of natural or semi-natural ecosystems, especially the plant community and soil chemistry, and soil microbiomes after cessation of N enrichment have been reported (10–14). Resistance and resilience are core components of ecosystem stability, which are defined as the ability of ecosystems to withstand perturbations and the ability of ecosystems to return to their original state after subsequent recovery, respectively (2, 15). In grassland ecosystems, knowledge gaps exist on the resistance of plant microbial communities to N deposition and their resilience to cessation of N addition.

The grassland ecosystems are distinguished by high biodiversity of plants, animals, and microbes. As an integral constituent of the grassland biodiversity, plant microbiomes have emerged as “the second genome” of plants (16) and play a pivotal role in upholding the diversity and functioning of the grassland ecosystems (17–21). A number of studies have attempted to utilize naturally occurring plant microbiota for developing sustainable agricultural systems (22–24) and regulating ecosystem functioning (25). Previous studies have revealed that host identity determined the bacterial and fungal communities and network structures in the phyllosphere of plant species in a temperate steppe (26). Recent studies also revealed that climate warming and N deposition increased leaf bacterial community diversity of a desert steppe (27). However, no study has specifically evaluated how the plant microbial community of the grassland ecosystem responds to global change factors in general and atmospheric N deposition and their legacy effects in particular.

Leaves and roots are major plant compartments colonized by a broad spectrum of microbes (28). The surface area of a leaf is about twice as that of the land surface (19). The composition of the microbial community differed markedly among plant compartments and varied in their responses to disturbance as they are exposed to distinct environments (24, 29, 30). For example, microbial communities in the phyllosphere and root endosphere of sorghum have been reported to be more resistant to long-term fertilization than soil microbiota (24). Recent studies also showed that the leaf-associated bacterial community was not resistant to mowing compared to the root-associated bacterial community in plant species of a temperate grassland (31). However, how leaf- and root-associated bacterial communities respond to long-term N addition and cessation of N addition in the temperate steppe has not been explored.

Here, we investigated the responses of plant microbial communities to consecutive N addition and cessation of N addition through a long-term field experiment in a temperate steppe of northern China. This experiment (i.e., 19 years of N addition followed by 6 years of cessation) allowed us to quantify the resistance of leaf- and root-associated bacterial communities to long-term N enrichment and their resilience following N cessation. In specific, we aimed to address the following three questions: i) Do N addition and N cessation influence leaf- and root-associated bacterial diversity and community structures? ii) Do the leaf- and root-associated bacterial diversity resistance and resilience respond differently to N addition and N cessation? iii) Which functional traits determine leaf- and root-associated bacterial diversity resistance and resilience in response to N addition and N cessation?

## RESULTS

### Alpha and beta diversity of leaf- and root-associated bacterial communities

**Fig 1:**
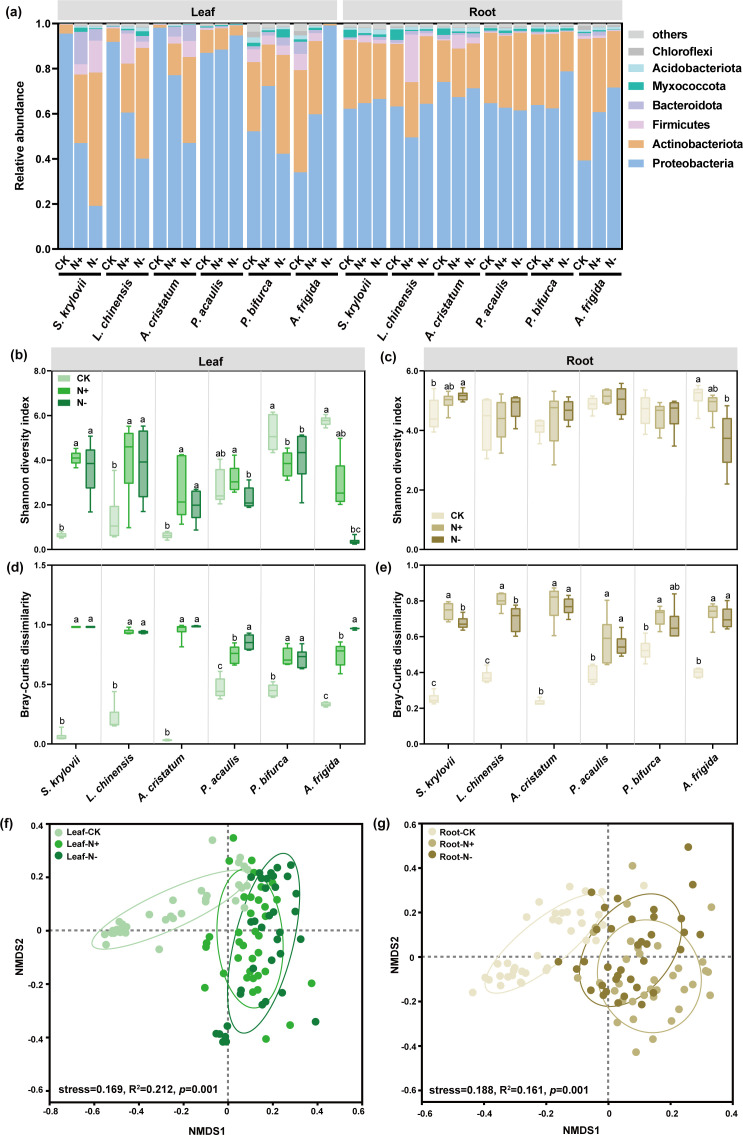
Effect of N addition and N cessation on the composition and α and β diversity of leaf- and root-associated bacterial communities. Relative abundance of leaf- and root-associated bacterial phyla (**a**). Shannon diversity index (α diversity) of leaf- and root-associated bacterial communities (**b and c**). Bray-Curtis dissimilarity (β diversity) of leaf- and root-associated bacterial communities (**d and e**). Box-and-whiskers plot illustrates the median (line inside the box), 25th and 75th quartiles (box ends), and maximum and minimum values (whiskers, *n* = 6). Different letters indicate significant differences among treatments at *P* < 0.05. Nonmetric multidimensional scaling (NMDS) analysis of the community composition of leaf- and root-associated bacterial communities (**f and g**). Ellipses in the plots represent the standard deviation of centroid means. Permutational multivariate analysis of variance (PERMANOVA) was performed for the factor of N treatment, and the analysis results are included in the images. CK, control; N+, N addition; N−, N cessation.

The bacterial communities across all the samples were dominated by the phyla Proteobacteria, Actinobacteriota, Firmicutes, and Bacteroidota (Fig. 1a). Nitrogen addition altered the distribution patterns of the dominant phyla of leaf-associated bacterial communities in the six individual plant species. However, the composition of root-associated bacterial communities for the six plant species exhibited a more stable pattern under N addition and cessation of N addition (Fig. 1a).

**Fig 2:**
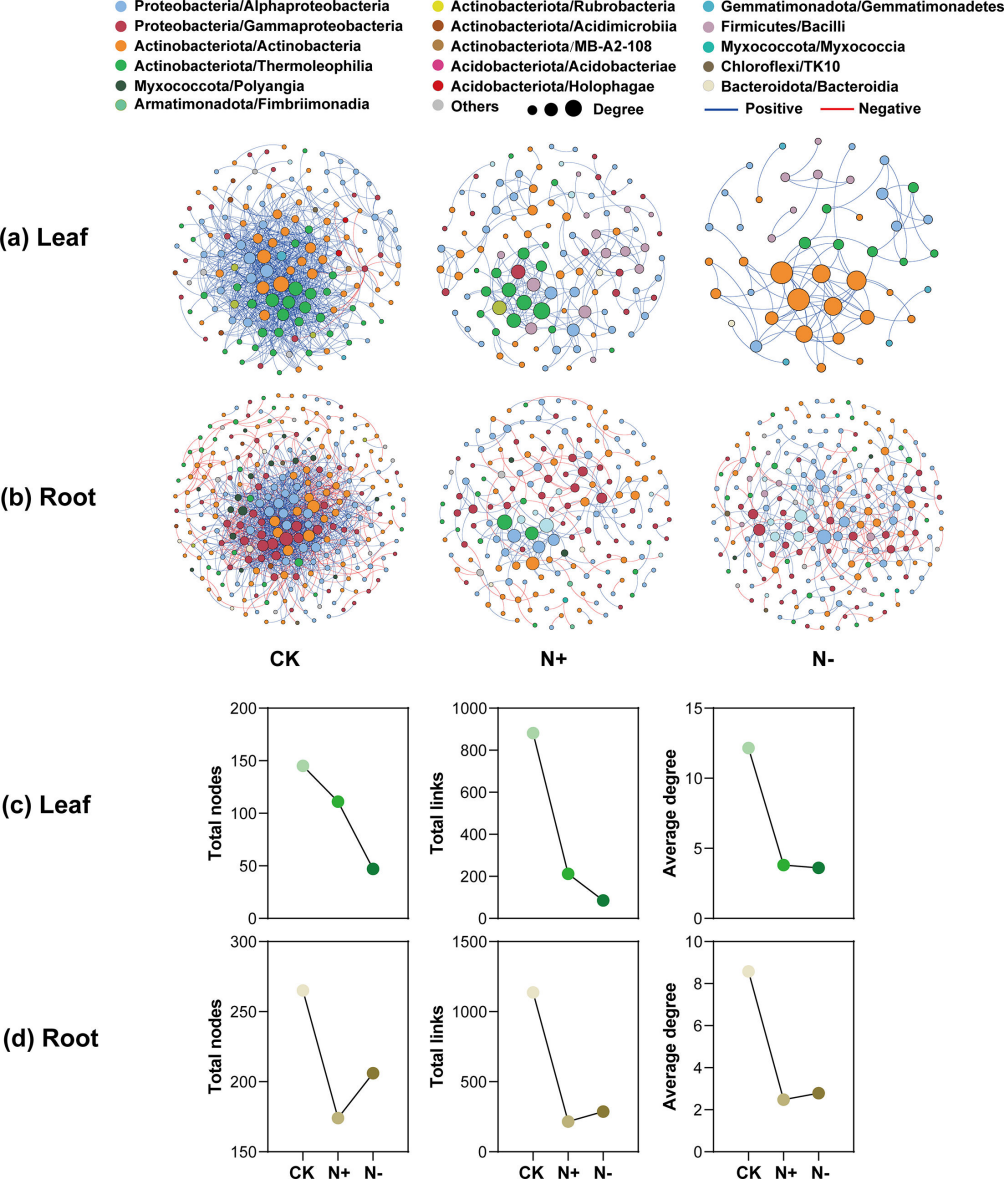
Effect of N addition and N cessation on co-occurrence network patterns. Co-occurrence networks of leaf- and root-associated bacterial communities at the class level under different treatments (**a and b**). Points in different colors denote different bacterial classes, and edges in blue and red colors represent positive and negative correlations, respectively. The size of a circle represents the node degree. Major topological properties of leaf- and root-associated bacterial communities under different treatments (**c and d**). CK, control; N+, N addition; N−, N cessation.

Nitrogen addition significantly influenced α diversity of leaf-associated bacterial communities in the sampled plant species (Fig. 2b). In contrast, N addition had no effect on the α diversity of root-associated bacterial communities in all the sampled plant species (Fig. 2c). After cessation of N addition, the α diversity of leaf- and root-associated bacterial communities had no significant recovery (Fig. 2b and c).

The leaf- and root-associated bacterial community composition in the N-addition plots diverged from the control plots (Fig. 1f and g), and the Bray-Curtis dissimilarity significantly increased with N addition (Fig. 1d and e). Results from permutational multivariate analysis of variance (PERMANOVA) showed that N addition had a significant effect on both leaf- and root-associated bacterial communities, with the effect being greater in the leaf-associated bacterial community than in the root-associated bacterial community for most of the individual plant species (i.e., *S. krylovii*, *L. chinensis*, *A. cristatum*, *P. bifurca*, and *A. frigida*) (Table S1). After cessation of N addition, the β diversity of leaf-associated bacteria had no significant recovery, while the β diversity of root-associated bacteria in *S. krylovii*, *L. chinensis,* and *P. bifurca* was significantly recovered, despite not fully recovering to the initial state in the control plots (Fig. 1d and e). As shown in the NMDS plot, the leaf-associated bacterial community under N addition and cessation of N addition was not separated (Fig. 1f). However, the root-associated bacterial community in the cessation of N addition exhibited a recovery trend, as evidenced by its position between the ambient and N addition (Fig. 1g). The effect of plant identity on the leaf- and root-associated bacterial communities was compared, and it was found that the effect sizes of plant species, family, and plant group were much greater on root-associated bacterial communities than on leaf-associated bacterial communities, both in N addition and cessation of N addition for all species (Table S2).

### Co-occurrence networks of leaf- and root-associated bacterial communities

Nitrogen addition led to reductions in the complexity of both leaf- and root-associated bacterial community networks, which was reflected by the decrease in the number of nodes and edges by N addition (Fig. 2a through d). After cessation of N addition, neither edges nor nodes of leaf-associated bacterial community were increased (Fig. 2c and d). In contrast, edges and nodes of root-associated bacterial community showed a certain degree of growth, indicating signs of recovery (Fig. 2c and d).

### Resistance and resilience of microbial diversity and relationships with plant and soil traits

**Fig 3:**
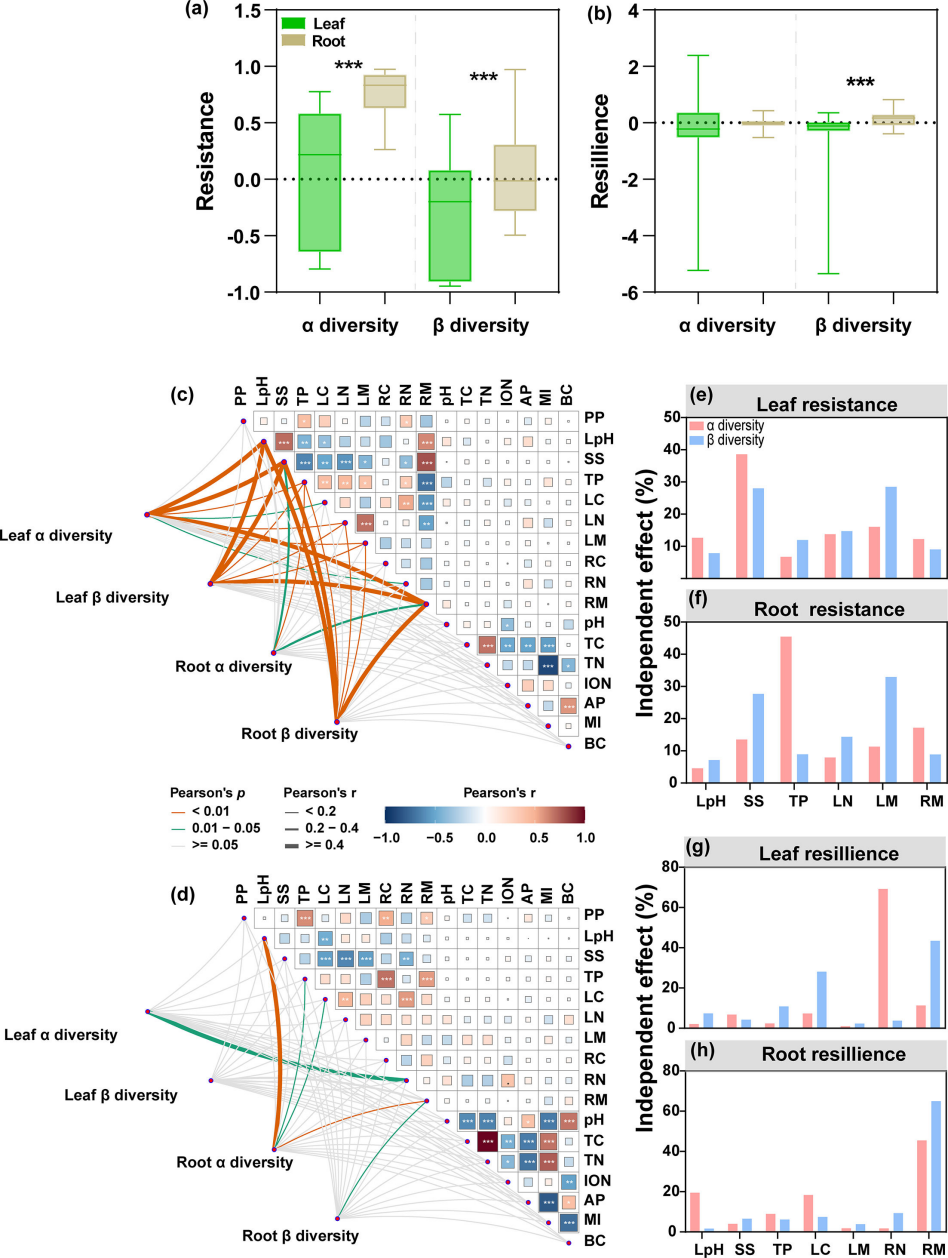
Resistance and resilience of bacterial diversity in response to N addition and N cessation and relationships with plant and soil traits. Resistance of α and β diversity in response to continuous N addition in leaf- and root-associated bacterial communities (**a**). Resilience of α and β diversity of leaf- and root-associated bacterial communities after N cessation (**b**). Diversity resistance correlated to changes in plant properties and soil chemical variables under N addition (**c**). Diversity resilience correlated to changes in plant properties and soil chemical variables under N cessation (**d**). Independent variance in plant properties attributed to resistance and resilience of leaf- and root-associated bacterial communities (**e, f, g, and h**). ***, *P* < 0.001. Plant properties include photosynthetic pigment (PP), leaf pH (LpH), soluble sugars (SS), total phenolics (TP), leaf carbon (LC), leaf nitrogen (LN), leaf morphology (LM), root carbon (RC), root nitrogen (RN), and root morphology (RM). Soil variables include pH, total carbon (TC), total nitrogen (TN), inorganic nitrogen (ION), available phosphorus (AP), metal ions (MI), and base cations (BC).

Both α and β diversity resistance in the root-associated bacterial community was found to be greater than that in the leaf-associated bacterial community for most of the individual plant species (Fig. 3a; Fig. S1a). The α diversity resilience of leaf- and root-associated bacterial communities did not differ significantly, whereas the β diversity resilience of the root-associated bacterial community was significantly higher than that of the leaf-associated bacterial community (Fig. 3b; Fig. S1b).

Pearson’s correlation analysis revealed that α- and β diversity resistance of leaf- and root-associated bacterial communities was significantly correlated with four leaf functional traits: leaf soluble sugars and total phenols, leaf N concentration, and leaf morphological traits (Fig. 3c). The hierarchical partitioning results revealed that the α- and β-diversity resistance of the leaf-associated bacterial community was mainly due to the changes in the concentrations of leaf soluble sugars and leaf morphology by N addition (Fig. 3e). Further, α- and β-diversity resistance of root-associated bacterial community mainly contributed to the changes in the concentration of soluble sugars, total phenols, and leaf morphology (Fig. 3f). In addition, changes in root morphology and root N content mainly explained the α and β diversity resilience in leaf- and root-associated bacterial communities (Fig. 3g and h).

### Functional groups of leaf- and root-associated bacterial communities

**Fig 4:**
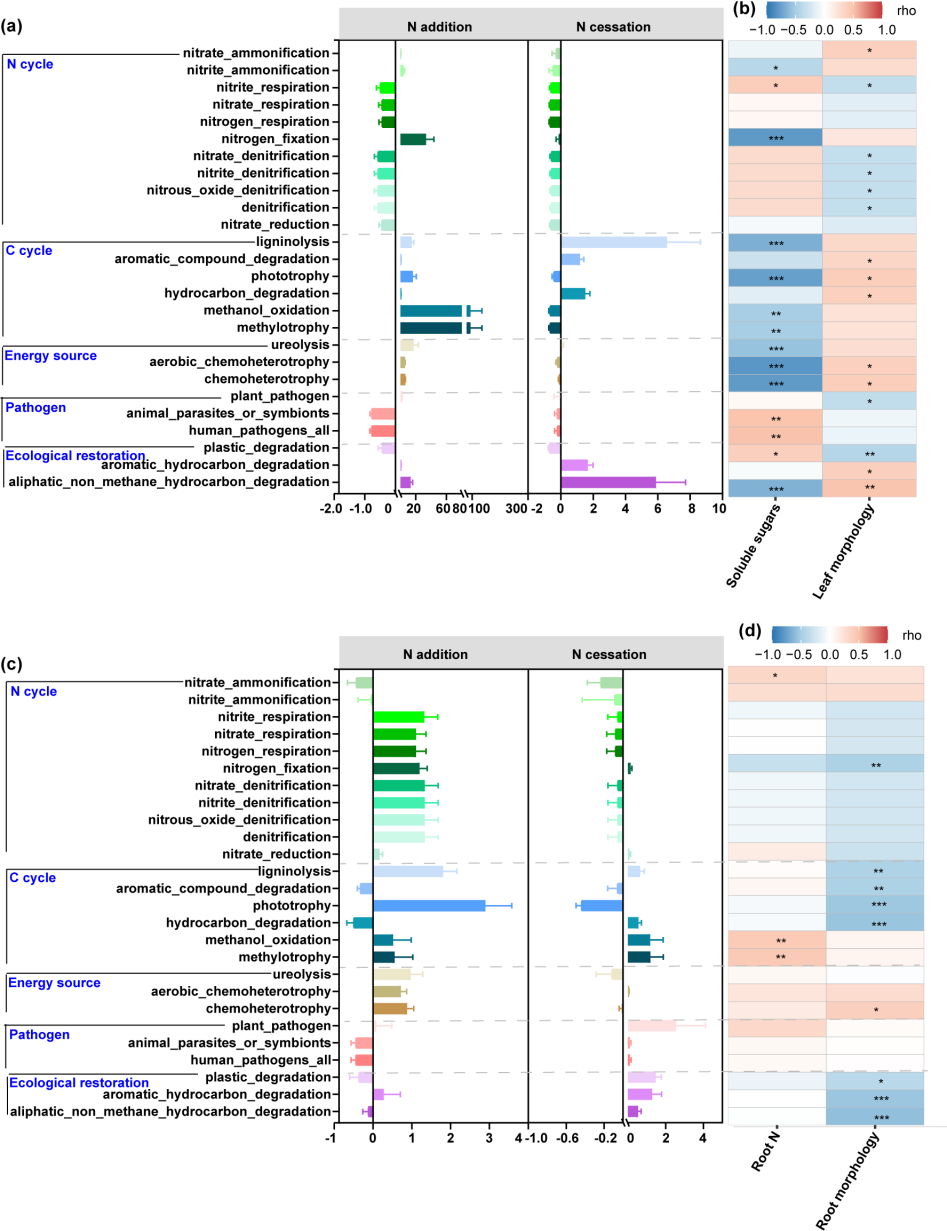
Abundance changes in bacterial functional groups and their correlations with soluble sugars, leaf morphology, and root N and root morphology. Abundance changes in the functional groups of leaf- and root-associated bacterial communities (**a and c**) under N addition and N cessation. Correlations between changes in the content of soluble sugars and leaf morphology and functional taxa of the leaf-associated bacterial community under N addition (**b**). Correlations between changes in the content of root N and root morphology and functional taxa of the root-associated bacterial community under N cessation (**d**). *, *P* < 0.05; **, *P* < 0.01; ***, *P* < 0.001.

For the leaf-associated bacterial community, N addition led to decreases in the relative abundance of bacteria associated with the N cycle (except for nitrogen fixation) and pathogens (Fig. 4a; Fig. S2). Bacterial functional groups involved in the C cycle, energy sources, and ecological restoration showed positive responses to N addition (Fig. 4a; Fig. S2). The cessation of nitrogen addition, however, resulted in limited recovery among functional groups of the leaf-associated bacterial community (Fig. 4a; Fig. S2). For the root-associated bacterial community, N addition led to increases in the relative abundance of bacteria associated with the N cycle (except for nitrate/nitrite ammonification), the C cycle (except for aromatic compounds and hydrocarbon degradation), and energy sources (Fig. 4c; Fig. S2). In addition, cessation of N addition recovered the relative abundance of bacterial functional groups associated with the N cycle, ecological restoration, and the C cycle (Fig. 4c; Fig. S2). Moreover, we found that the abundance change of functional groups in the leaf-associated bacterial community induced by N addition was significantly influenced by variation in leaf structure and soluble sugars (Fig. 4b). The recovery of functional groups in the root-associated bacterial community was significantly influenced by variation in root N and root morphology (Fig. 4d).

### Potential sources

**Fig 5:**
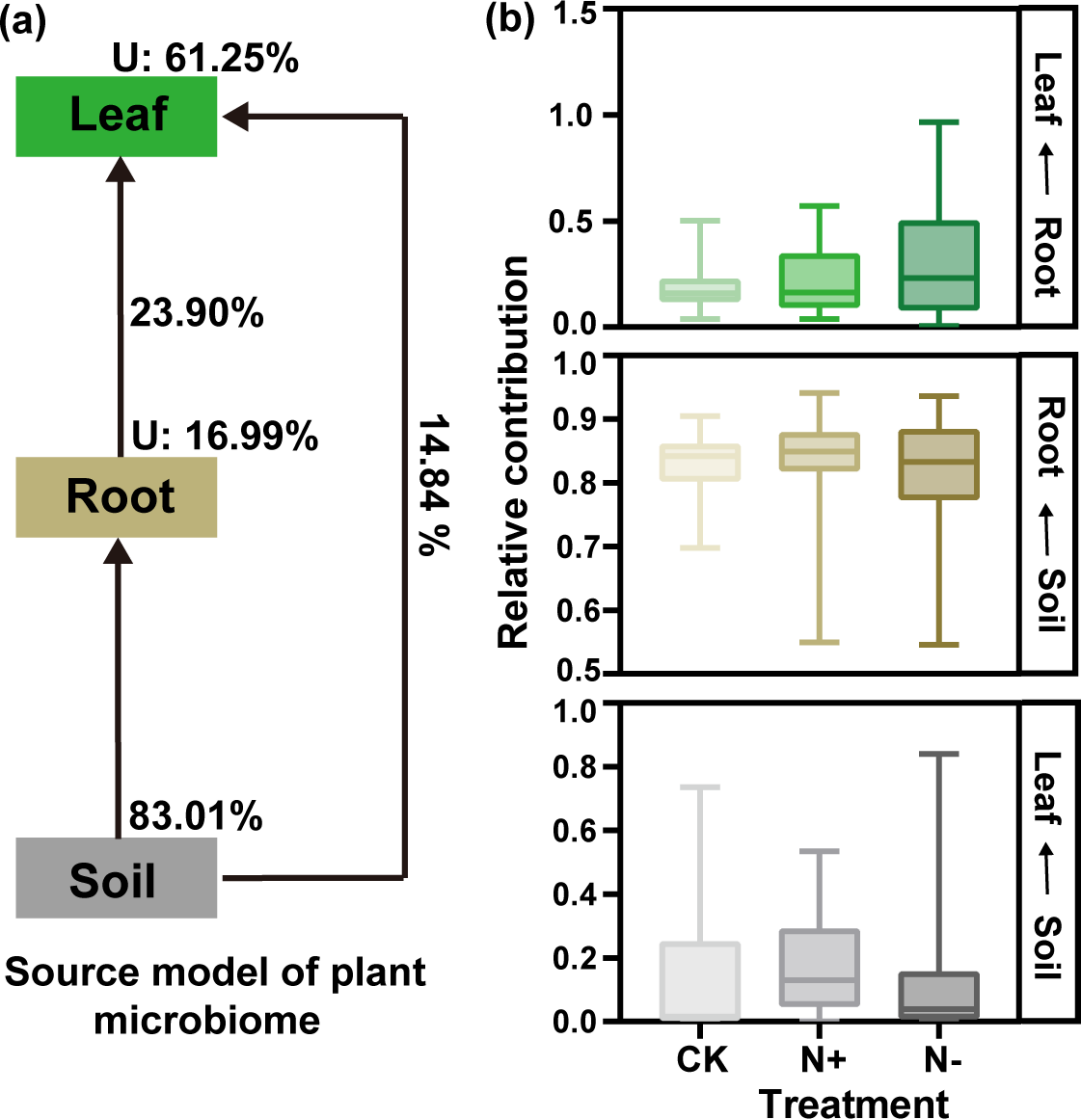
Source model of plant microbiome. Source model showing potential sources of leaf- and root-associated bacterial communities (**a**). U stands for unknown source. Relative contributions of root-associated and soil bacterial communities to sources of the leaf-associated bacterial community and relative contributions of the soil bacterial community to sources of the root-associated bacterial community under control, N addition, and N cessation (**b**). Box-and-whiskers plot illustrates the median (line inside box), 25th and 75th quartiles (box ends), and maximum and minimum values (whiskers). CK, control; N+, N addition; N−, N cessation.

Results from source-tracking analysis revealed that plant-associated bacterial communities were mainly derived from bulk soil and gradually filtered by different plant compartmental niches (Fig. 5a). In addition, the relative contributions of sources to leaf- and root-associated bacterial communities were not influenced by N addition and cessation of N addition (Fig. 5b).

## DISCUSSION

Our study revealed that leaf- and root-associated bacterial communities differed in their responses to N disturbance in a temperate grassland. The leaf-associated bacterial community exhibited lower resistance to N enrichment, and the resistance was mainly steered by leaf soluble sugars and leaf morphological traits. The unique environment of the phyllosphere may render the leaf-associated bacterial community with lower resistance to the fluctuation of the environment. The root-associated bacterial community showed stronger resilience than the leaf-associated bacterial community. This may be attributed to the restored root N and root morphology. The greater stability in the root-associated bacterial community may also be ascribed to a higher presence of host-related factors. In addition, we found that N addition-induced suppression of beneficial symbiotic microbes involved in N cycle in the leaf-associated bacterial community was hardly recovered after cessation of N addition. In contrast to the leaf-associated bacterial community, N addition-induced inhibition of microbes involved in C cycle in the root-associated bacterial community displayed a quick recovery after cessation of N enrichment. These results offer valuable insights into the impacts of alterations in N input on resistance and resilience of two key plant microbial communities, leaf- and root-associated bacterial communities.

We explored the responses of leaf- and root-associated bacterial communities to enhanced N input based on a long-term manipulative N-addition experiment in a temperate grassland of northern China. Given their distinct growing environments (i.e., aboveground and belowground for leaf- and root-associated bacterial communities, respectively) and the great differences in the structure and physiochemical properties between leaves and roots (24, 30, 32), we speculate that leaf- and root-associated bacterial communities would display different responses to N addition. Consistent with this speculation, we observed that leaf- and root-associated bacterial communities differed in their responses to long-term N addition in the temperate grassland. This observation can be explained by the fact that Shannon diversity index and the relative abundance of the dominant phyla of leaf-associated bacterial communities are less resistant to long-term N addition than root-associated bacterial communities. These results may collectively suggest that the leaf-associated bacterial community is less resistant to N addition than the root-associated bacterial community in the temperate grassland. This argument is also corroborated by the higher resistance index of alpha and beta diversities of root-associated bacterial than leaf-associated bacterial communities (Fig. 3a; Fig. S1a). A recent study reported that the phyllosphere microbiome is less resistant to long-term mowing than the root microbiome in the same temperate grassland (31). In addition, microbial functional traits in the phyllosphere are found to be less resistant to anthropogenic disturbance than in soil (32). These results indicate that the structure of the leaf-associated bacterial community may be equipped with lower resistance than that of the root-associated bacterial community when temperate grasslands are challenged by disturbances.

Nitrogen addition reduced the complexity of leaf- and root-associated bacterial networks, which was attributed to the decreased nodes and edges (Fig. 2). Moreover, the number of nodes and edges in the root-associated bacterial community co-occurrence network was significantly higher than that in the leaf-associated bacterial community networks in different treatments, indicating that the root-associated bacterial community network is more complex than the leaf-associated bacterial network (Fig. 2a and b). This finding is consistent with the results showing that the root-associated bacterial community network is more complex than the leaf-associated bacterial network under both control and long-term mowing treatments (31). Similar to grassland ecosystems, the bacterial network complexity sequentially decreased along the soil-plant continuum in agricultural ecosystems (33). The fungal networks in the rhizosphere of *Bothriochloa ischaemum*, which is a dominant plant species in copper tailings dams, are more complex than those in the phyllosphere (34). These results highlight that the network complexity of microbial communities in belowground plant compartments is much greater than that in aboveground plant compartments.

Environmental changes can evoke great variability in plant traits (35), thus altering the plant-associated microbial community composition (36). Therefore, it is conceivable that the resistance of the bacterial community to N enrichment would be closely linked to changes in plant morphological and physiochemical traits. In consistence with this expectation, we found that the foliar concentrations of soluble sugars, total phenols, N, and leaf morphological traits were strongly correlated with α and β diversity resistance of the leaf-associated bacterial community. Hierarchical partitioning analysis further confirmed the independent contribution of these functional traits to the diversity resistance of the leaf-associated bacterial community, with foliar soluble sugars and leaf morphological traits being the top two contributors. This observation is consistent with those of previous studies demonstrating that the diversity and structure of the phyllosphere microbial community are regulated by leaf soluble sugars. For instance, bacterial community structure in the phyllosphere of *Lactuca* species was regulated by soluble sugars and their derivatives (37). Similarly, sugars that are major carbon-containing compounds on the leaf surface of plant species are a key determinant of the population of epiphytic bacteria (38). The close correlation between soluble sugars and microbial community could be explained by their roles as carbon sources for microbes. Leaf morphology, which may have the potential to influence the leaf microenvironment and topographical features, is another important driving factor determining the microbial community (37). This could explain the strong relationship between resistance of leaf-associated bacterial community diversity and leaf morphological traits. We further illustrated that foliar soluble sugars and leaf structure were involved in the regulation of leaf-associated bacterial communities in response to N addition by differently influencing bacterial functional groups (Fig. 4b). Consequently, leaf soluble sugars and morphological structure may steer the diversity resistance of the leaf-associated bacterial community by shaping their functional groups.

Results from some agricultural, forest, and model plant species have suggested that root traits selectively filter and recruit different microbial communities (39). The combination of Pearson’s correlation analysis and hierarchical segmentation analysis revealed that the recovery of root N content and root morphological traits exerted a significant influence on the resilience of root-associated bacterial community. Our results are supported by those of a recent study demonstrating that the root-associated bacterial diversity was mainly correlated with root trait variation (31). In addition to bacteria, root traits, particularly root N content and specific root length, are also significant factors in shaping the composition of rhizosphere fungal communities (36). Our study further illustrated that root N and morphology were involved in regulation of the root-associated bacterial community in response to the cessation of N addition by influencing bacterial functional groups (Fig. 4d). Consequently, root N and root morphology may steer the diversity resilience of the root-associated bacterial community by shaping their functional groups. Unfortunately, we were unable to identify the key drivers behind the restoration of functional groups associated with the N cycle. In this study, only root C, root N, and root morphological traits were measured. It is possible that the resilience of root-associated bacterial community diversity may also be related to other root traits rather than root C, root N, and root morphological traits. The close association between root exudates and microbial community structure has been reported (40), thus necessitating their inclusion in future studies.

Another interesting finding is that the effect sizes of plant species, family, and plant group are much greater in the root-associated bacterial community than in the leaf-associated bacterial community under both N addition and cessation of N addition conditions, which may imply that host plants have a greater impact on the structure of the root-associated bacterial community than on the leaf-associated bacterial community. Root traits have been suggested to depend more on the host plant than leaf traits based on large-scale studies (31). Therefore, the greater influence of host plants on the root-associated bacterial community than on the leaf-associated bacterial community may be related to species-specific root traits (41, 42). These results highlight the dominant roles of plant species identity in modulating belowground root systems and the root-associated bacterial community.

Plant-microbe interactions are often bidirectional: microbial communities can also influence root morphology, nutrient uptake efficiency, and stress tolerance (20, 43). In addition, soil microbial legacy effects under long-term N enrichment may partly structure RAB communities independently of plant traits. We cannot determine the causal direction between plant and microbe interactions here, and plant traits and microbiomes may co-respond to shared environmental drivers.

The plant-associated bacterial community was mainly derived from bulk soil and gradually filtered by different plant compartment niches (31, 33, 44). In the present study, we also found that the root-associated bacterial community was mainly determined by the bulk soil (Fig. 5a). However, the proportion of the leaf-associated bacterial community with unknown sources was up to 61.25% (Fig. 5a). The phyllosphere is considered a more nutrient-limited environment and exposed to more variable conditions than the rhizosphere, such as fluctuations in temperature, moisture, and UV radiation (19, 45). The relative contributions of other sources like air, splashing rain, crawling insects, or seeds to the composition of the phyllosphere community may influence the fluctuation of the leaf-associated bacterial community (46), which warrants further investigation in future studies. The unique environment of the phyllosphere may render the leaf-associated bacterial community lower in resistance to environmental fluctuations, whereas the root system is predominantly influenced by the host, resulting in greater resilience in the root-associated bacterial community.

### Conclusions

Based on a 19-year field experiment in a temperate steppe, we discovered that leaf- and root-associated bacterial communities respond differently to N disturbance in a temperate grassland. Leaf-associated bacterial communities exhibit lower resistance to N enrichment, and the resistance is mainly steered by soluble sugar and leaf morphology through regulation of the functional taxa. Root-associated bacterial communities demonstrate stronger resilience. This may be attributed to the restored root N and root morphology regulating functional taxa. Our study offers valuable insights into the impact and mechanisms of N interference on plant microbial communities. Such an understanding is crucial for guiding grassland management strategies aimed at preserving functional microbial communities and maintaining grassland biodiversity amid ongoing global change.

## MATERIALS AND METHODS

### Experimental design

The N addition experiment was conducted in a temperate steppe in Duolun County, Inner Mongolia Autonomous Region, China (116^o^17′ E, 42^o^02′N; 1,324 m a.s.l.). The experimental site has a mean annual temperature of 2.1°C and a mean annual precipitation of 385.5 mm. Soil in this area is chestnut soil with a pH of approximately 7.0 and a bulk density of 1.31 g cm^−3^. The dominant plant species in this grassland are *Stipa krylovii*, *Agropyron cristatum*, *Leymus chinensis*, *Artemisia frigida, Potentilla bifurca,* and *Potentilla acaulis* (Fig. S3). The experiment was set up in 2003 after exclusion of livestock grazing by fencing. There were six levels of N addition (0, 2, 4, 8, 16, and 32 g N m^−2^ year^−1^), with six plots for each N-addition level. The plot was 15×10 m in size and separated by 4-m-wide buffer strips. Urea (N, 46%) was added evenly to each plot to simulate N deposition in June and July when maximal precipitation occurred. In 2016, the N-added plots were divided into two sub-plots equally by a steel plate (30 cm depth). Nitrogen addition was ceased in one of the sub-plots, while N was continuously added in the other sub-plot, as described previously (10).

### Sample collection and measurement

In early August 2022, we collected soils and plants from 18 plots (six replicates) supplemented with two levels of N (0 and 8 g N m^−2^ year^−1^) and two types of N treatment (consecutive N addition and cessation of N addition). In this region, the level of current natural N deposition is 1.5 g N m^−2^ year^−1^. In this study, we selected plots with ambient N deposition and N addition at a rate of 8 g N m^−2^ year^−1^ for both the N addition and controls. This N-addition level is higher than the current natural N deposition rate in this region, but atmospheric N deposition rates as high as 5.5 g N m^−2^ year^−1^ in China (47) and 3.6 g N m^−2^ year^−1^ in the United Kingdom (48) have been reported. Moreover, N-addition rates up to 9.5 g N m^−2^ year^−1^ (49), 14.0 g N m^−2^ year^−1^ (50), and 10.0 g N m^−2^ year^−1^ (51) have been used in N-addition experiments of grasslands in North America, Europe, and China, respectively. Six perennial herbaceous species belonging to three families and two plant functional groups were sampled (Table 1). For each plant species, we randomly dug soil blocks (30 × 30 × 30 cm, length, width, and depth, respectively) around the target plants to collect intact plants and sufficient leaf/root tissues. All roots were collected with the block for measurements of root traits and extraction of DNA. Green leaves were collected from the plants that were dug in the block. Topsoil (0–10 cm) was collected using an auger. Three soil cores were pooled into one soil sample and sieved through a 2-mm mesh in each plot. In total, we obtained 234 samples, including 108 leaf samples, 108 root samples, and 18 soil samples. These samples were shipped in ice boxes, and the samples for DNA extraction were kept frozen at −80°C in the laboratory.

**TABLE 1:** Information of the six selected plant species in the temperate steppe

Species	Abbreviation	Family	Plant group
*Stipa krylovii*	*S. krylovii*	Poaceae	Grass
*Leymus chinensis*	*L. chinensis*	Poaceae	Grass
*Agropyron cristatum*	*A. cristatum*	Poaceae	Grass
*Potentilla acaulis*	*P. acaulis*	Rosaceae	Forb
*Potentilla bifurca*	*P. bifurca*	Rosaceae	Forb
*Artemisia frigida*	*A. frigida*	Asteraceae	Forb

The plant traits related to plant growth status, morphology, and physiological processes, which have been frequently used in previous studies, were measured (31, 46, 52). Specifically, leaf traits including concentrations of foliar chlorophyll, carotenoid, soluble sugars, total phenolics, leaf pH, leaf area, specific leaf area, leaf water content, leaf dry matter content, leaf total nitrogen, and leaf total carbon were measured (Fig. S4). Root traits including specific root length, specific root area and root tissue density, root total nitrogen, and root total carbon were determined (Fig. S5). In addition, the soil characteristics, including soil pH, soil total carbon, soil total N, nitrate-N (NO_3_^−^-N), ammonium-N (NH_4_^+^-N), soil available phosphorus (Olsen-P), metal ions (exchangeable Fe^3+^, Mn^2+^, Al^3+,^ and Cu^2+^), and base cations (K^+^, Ca^2+,^ and Mg^2+^), were also quantified (Fig. S6). The detailed methods for leaf trait measurement were previously described (7), and the methods for root trait and soil characteristics measurement can be found in Method S1.

### DNA extraction, bacterial 16S rRNA gene amplification, and sequencing

Green and healthy leaves were directly used for isolation of DNA, while roots were washed with sterile water to remove soils attached to the root surface and then used for isolation of DNA (44). Leaf/root epiphytic and endophytic microbiomes were not separated in this study (44). About 0.5 g of fresh leaf and root samples and 0.3 g of fresh soil were used to isolate genomic DNA with the FastDNA SPIN Kit (MP Biomedicals), following the manufacturer’s instructions. Bacterial variable region V5–V7 of the 16S rRNA gene was amplified using primer pair 799F/1392R (799F: 5′-AACMGGATTAGATACCCKG-3′; 1392R: 5′-ACGGGCGGTGTGTRC-3′), followed by primer pair 799F/1193R (1193R: 5′-ACGTCATCCCCACCTTCC-3′) (20, 43, 53, 54). The two-stage PCR approach has been previously used to prepare amplicon libraries in studies of phyllosphere bacterial communities (53, 55). The amplicons were sequenced using the Illumina MiSeq PE300 platform (Illumina, San Diego, CA, USA).

The raw sequencing reads were quality-filtered by Trimmomatic and merged by FLASH, as previously described (56, 57). The reads were truncated at any site receiving an average quality score less than 20 over a 50-bp sliding window. The truncated reads shorter than 50 bp or containing ambiguous characters were removed, and the sequences of the overlapping region longer than 10 bp were assembled. The mismatch ratio of the overlapping region was less than 0.2. The sequences of each sample were distinguished according to barcodes (exact matching) and primers allowing two nucleotide mismatches. Sequences were clustered into operational taxonomic units (OTUs) at 97% similarity cut-off using UPARSE version 7.1 with a novel “greedy” algorithm (58). The annotation of each OTU representative sequence was conducted by blasting against the SILVIA database with a confidence threshold of 0.7 (59). OTUs with less than 10 reads were discarded from all samples to reduce PCR/sequencing errors (60). OTUs identified as “chloroplast” and “mitochondria” were excluded. There were a total of 6,316,310 sequences for the bacterial community across the 234 samples. The number of bacterial sequences per sample was rarefied to 11,811. After rarefaction, the sequences were further clustered into 5,572 bacterial OTUs at 97% sequence similarity (Fig. S8).

### Statistical analyses

For the analysis of diversity, resistance, and resilience, if the data meet the requirements of normality and homoscedasticity of residuals, paired-samples *t*-test was conducted; otherwise, Wilcoxon Signed Rank test was performed. For the analyses of leaf- and root-associated bacterial Shannon index, Bray-Curtis dissimilarity, and plant and soil traits, if the data meet the requirements of normality and homoscedasticity of residuals, one-way ANOVA was performed; otherwise, Kruskal-Wallis test was performed. Nonmetric multidimensional scaling (NMDS) was conducted to display bacterial community dissimilarities with the metaMDS function in the vegan package (61). Effects of factors on bacterial community dissimilarities were evaluated by permutational multivariate analysis of variance (PERMANOVA) using the “adonis” function in the vegan package. For each factor (treatment, species, family, and plant group), PERMANOVA was conducted separately.

The Shannon diversity index (*H*) and Bray-Curtis dissimilarity (Dis) were used to determine community α and β diversity, respectively. The *H* and Dis of the bacterial communities were obtained using the data of OTUs by the vegan package. To quantify the effects of N addition on bacterial diversity, we defined diversity resistance (*V*_*r*_) by comparing the differences in bacterial community diversity between control and N addition. The α diversity resistance was calculated using this equation (62).


(1) $$V_{r(\alpha )}=1-\left(\frac{2|H_{\left(N+\right)}-H_{\left(CK\right)}|}{H_{\left(CK\right)}+|H_{\left(N+\right)}-H_{\left(CK\right)}|}\right)$$

where *H*_(N+)_ and *H*_(CK)_ are the values of the Shannon diversity index under N addition and control treatments, respectively. The β diversity resistance, *V*_*r*(β)_, was calculated as follows:


(2) $$V_{r(\beta )}=1-\left(\frac{2|{\text{Dis}}_{\left(N+\right)}-{\text{Dis}}_{\left(CK\right)}|}{{\text{Dis}}_{\left(CK\right)}+|{\text{Dis}}_{\left(N+\right)}-{\text{Dis}}_{\left(CK\right)}|}\right)$$

where Dis_(N+)_ is the Bray-Curtis distance of replicate bacterial communities within N addition plots to control and Dis_(CK)_ is the mean Bray-Curtis distance of replicate communities in control plots. The resistance index is bounded by −1 and 1, with the value of 1 indicating that N addition has no effect (maximal resistance) and lower values indicating less resistance. The resilience was used to evaluate the responses of bacterial communities to the cessation of N addition by comparing the relative differences between cessation of N addition and consecutive N addition relative to control. The α and β diversity resilience were calculated using these equations.


(3) $$V_{E(\alpha )}=\frac{H_{\left(N-\right)}-H_{\left(N+\right)}}{H_{\left(CK\right)}}$$

(4) $$V_{E(\beta )}=\frac{{\text{Dis}}_{\left(N+\right)}-{\text{Dis}}_{\left(N-\right)}}{{\text{Dis}}_{\left(CK\right)}}$$

where *H*_(CK)_, *H*_(N+)_, and *H*_(N−)_ are the values of community *H* within control, continuous N addition, and cessation of N addition, respectively; Dis_(N+)_ is the Bray-Curtis distance between the continuous N addition and control; and Dis_(N−)_ is the Bray-Curtis distance between the cessation of N addition and control; and Dis_(CK)_ is the mean Bray-Curtis distance of replicate communities in control plots.

Leaf area, specific leaf area, leaf dry matter content, and total water content were categorized into leaf morphology, while specific root length, specific root area, and root tissue density were categorized into root morphology. Thereafter, principal component analysis (PCA) was performed for each category, and the first principal component (PC1) was used to represent each variable category (i.e., leaf morphology and root morphology). The relationship between diversity resistance and resilience and plant/soil variables was determined by Pearson’s correlation analysis. Hierarchical partitioning was conducted to determine the independent contribution of plant variables to the diversity resistance and resilience in the “hier.part” package. Functional annotation of bacterial taxa was performed using “FAPROTAX” (63). Spearman’s rank correlation analysis was used to evaluate the relationship between changes in the relative abundance of bacterial functional taxa and alterations in soluble sugar, leaf morphology, root N, and root morphology.

To assess the effects of N addition and cessation of N addition on the network complexity and co-occurrence patterns of leaf-associated bacterial/root-associated bacterial taxa, network analysis was performed in the integrated Network Analysis Pipeline (64, 65). The network complexity was defined following protocols as described previously (66). The resultant correlations of the co-occurrence network were visualized by the Gephi software (v. 0.9.2) (67). To characterize their enrichment processes along the soil-plant continuum by the concept model (44), the Fast Expectation-mAximization microbial Source Tracking (FEAST) program (68) was used to determine the proportions of leaf- and root-associated bacterial communities sourced from different compartments. Root-associated bacterial and soil bacterial communities were set as the sources of leaf-associated bacterial communities, while soil bacterial communities were set as the source of root-associated bacterial communities (33). All statistical analyses were performed in R software (version 4.2.2, R Core Team, 2022).

## Data Availability

The sequencing data were deposited in the NCBI Sequence Read Archive under accession numbers PRJNA1422422 and PRJNA1336946.

## SUPPLEMENTAL MATERIAL

Supplemental material aem.00332-26-s0001.docx Figures S1 to S8, Tables S1 and S2, and Methods S1.

ASM does not own the copyrights to Supplemental Material that may be linked to, or accessed through, an article. The authors have granted ASM a non-exclusive, world-wide license to publish the Supplemental Material files. Please contact the corresponding author directly for reuse.
