# Supplementary material for: Leaf- and root-associated bacterial communities differ in their resistance and resilience to N disturbance in a temperate steppe
Source: Appl Environ Microbiol. 2026 May 27;92(6):e00332-26. doi: 10.1128/aem.00332-26 (PMC13289719; doi:10.1128/aem.00332-26)
Supplement: Supplemental material — Figures S1 to S8, Tables S1 and S2, and Methods S1. [file aem.00332-26-s0001.docx]

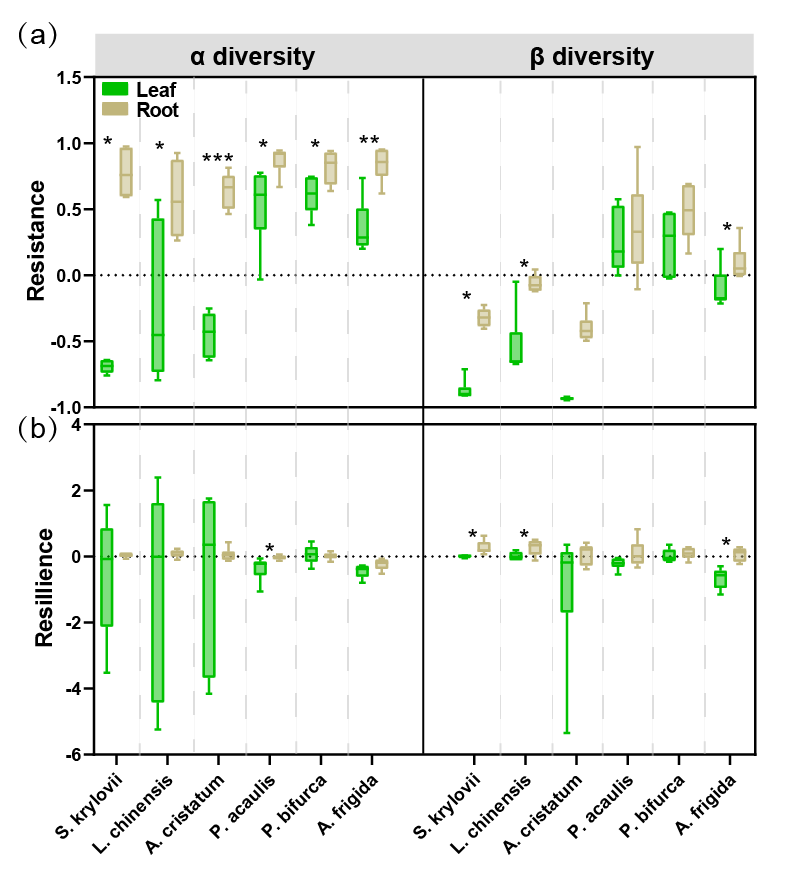


**Fig. S1.** Resistance and resilience of bacterial diversity in response to nitrogen (N) addition and N cessation. Resistance of α and β diversity in response to continuous N addition in leaf- and root-associated bacterial communities of different plant species (a). Resilience of α and β diversity of leaf- and root-associated bacterial communities after cessation of N addition of different plant species (b).


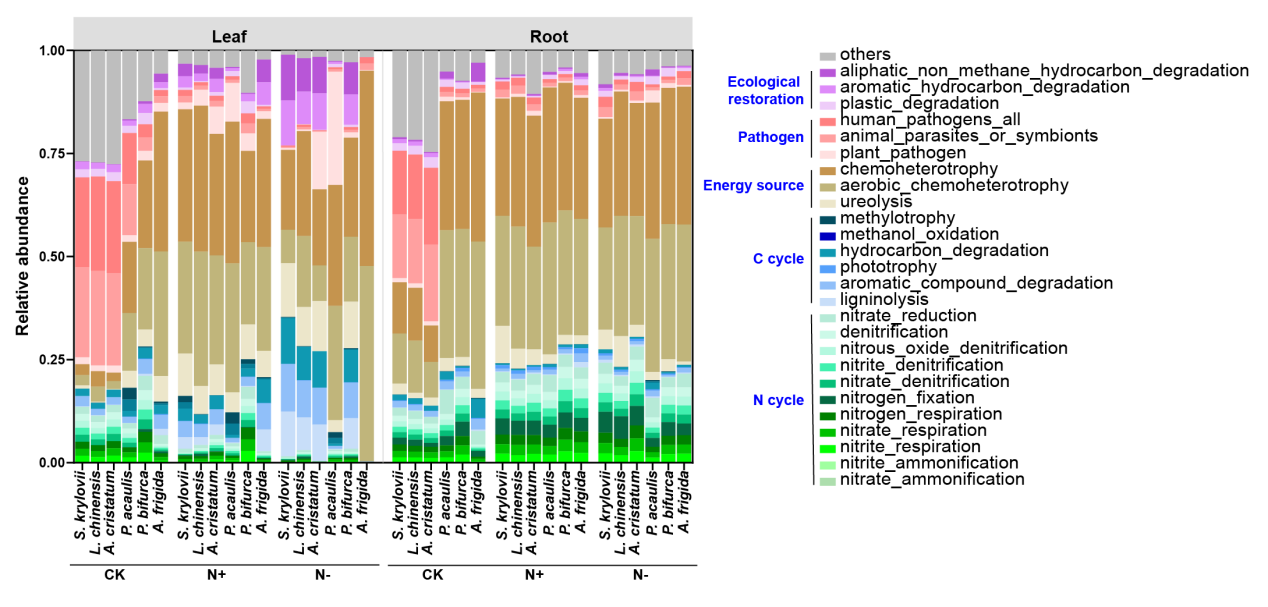


**Fig. S2.** Effect of N addition and N cessation on the relative abundance of leaf- and root-associated bacterial functional groups of different plant species.

**
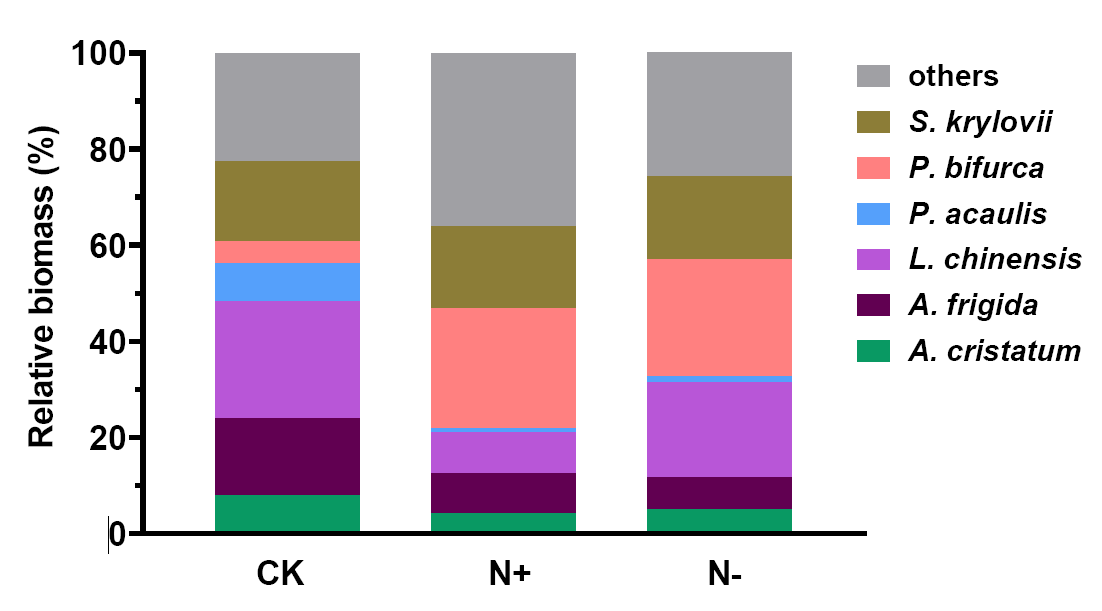
**

**Fig. S3** Plant community composition under different treatments. “others” indicates the total relative biomass of the remaining plants species in the plots. CK, control; N+, N addition; N-, N cessation.

**
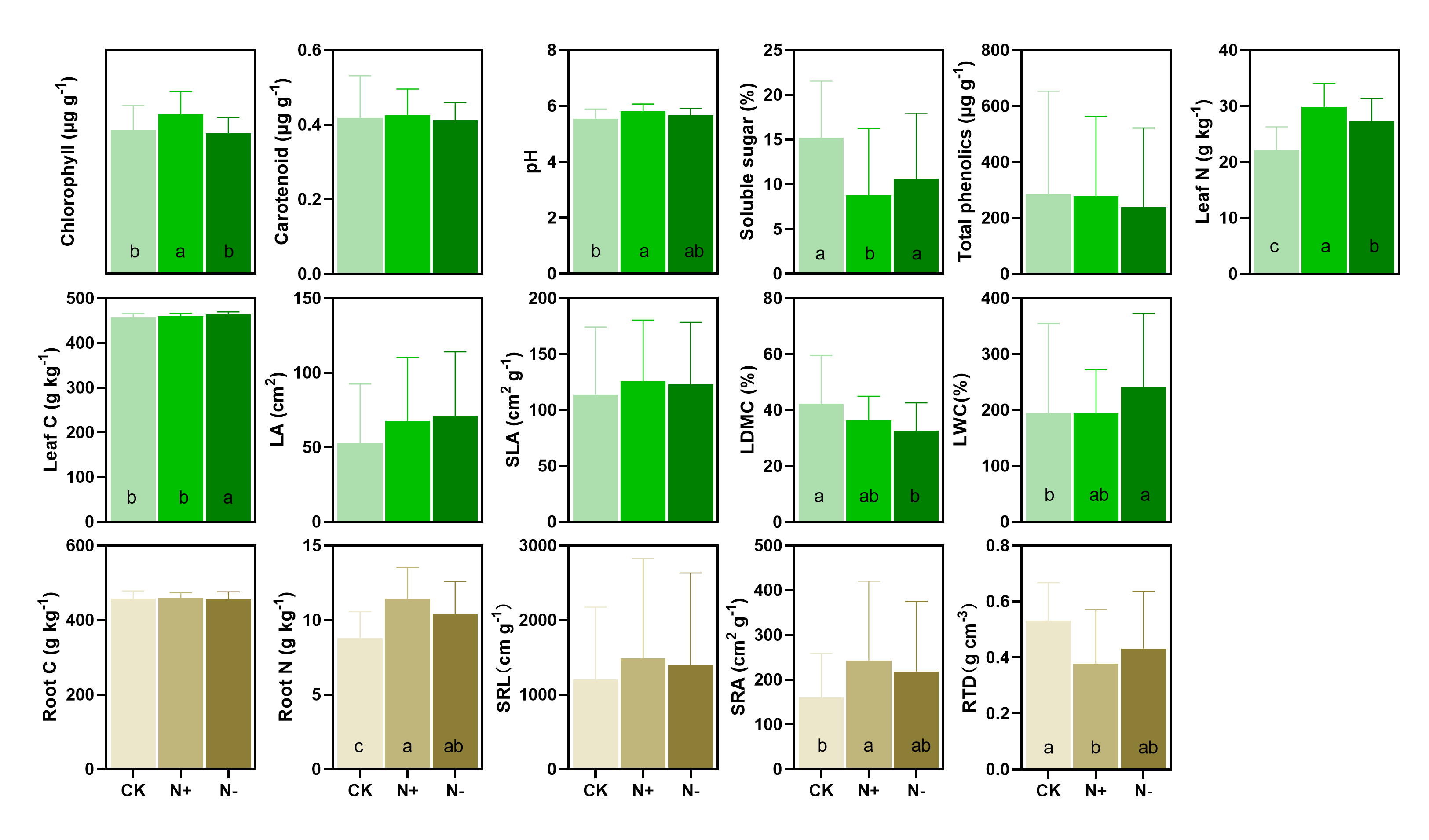
**

**Fig. S4** Effects of N addition and N cessation on leaf and root variables. Data are means ± SD. CK, control; N+, N addition; N-, N cessation. Different letters indicate significant difference among treatments at p < 0.05. LA, leaf area; SLA, specific leaf area; LDMC, leaf dry matter content; LWC, leaf water content; SRL, specific root length; SRA, specific root area; RTD, root tissue density.


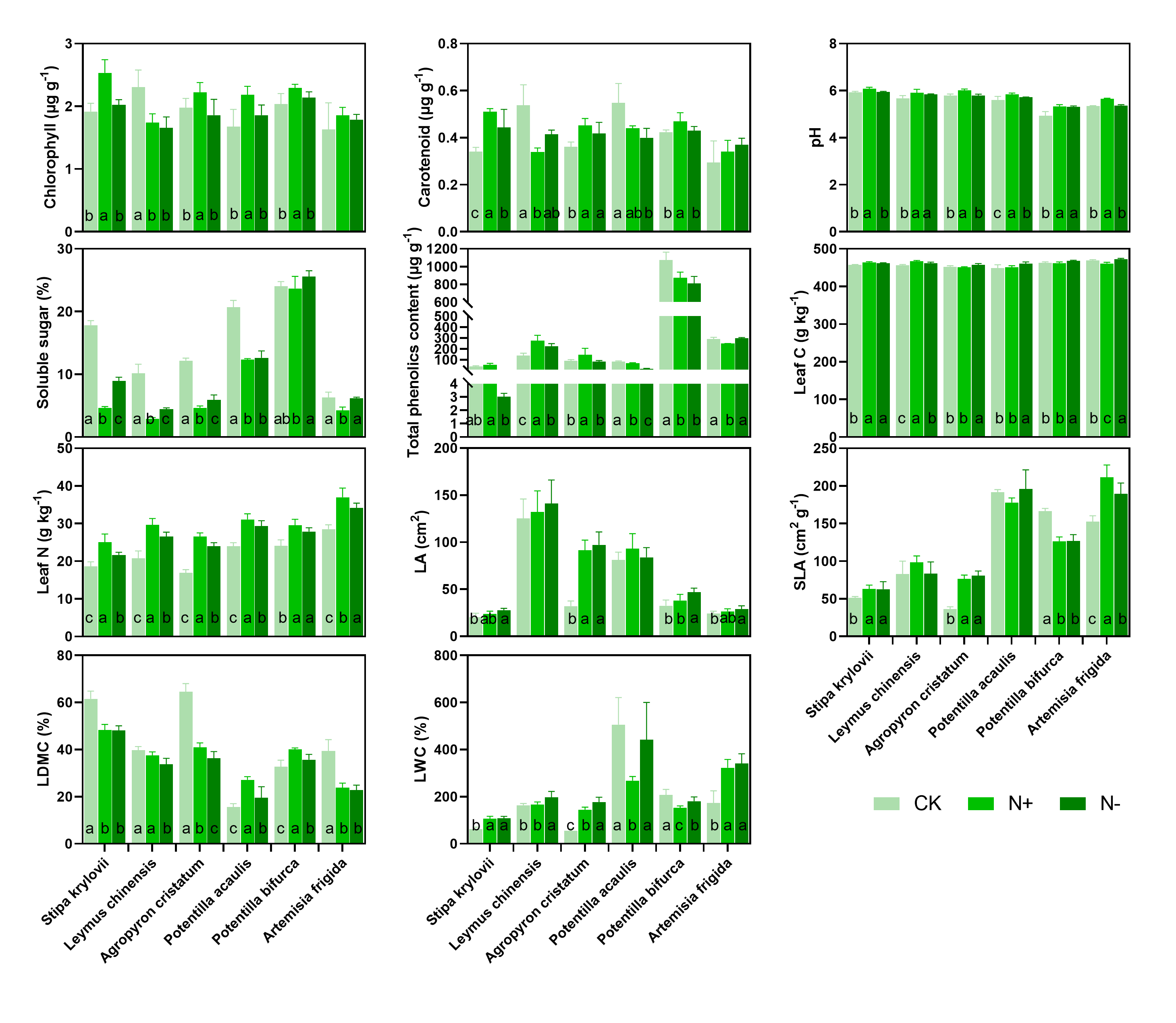


**Fig. S5.** Effects of N addition and N cessation on leaf variables of different plant species. Data are means ± SD for six replicates. CK, control; N+, N addition; N-, N cessation. Different letters indicate significant difference among treatments at *p* < 0.05. LA, leaf area; SLA, specific leaf area; LDMC, leaf dry matter content; LWC, leaf water content.


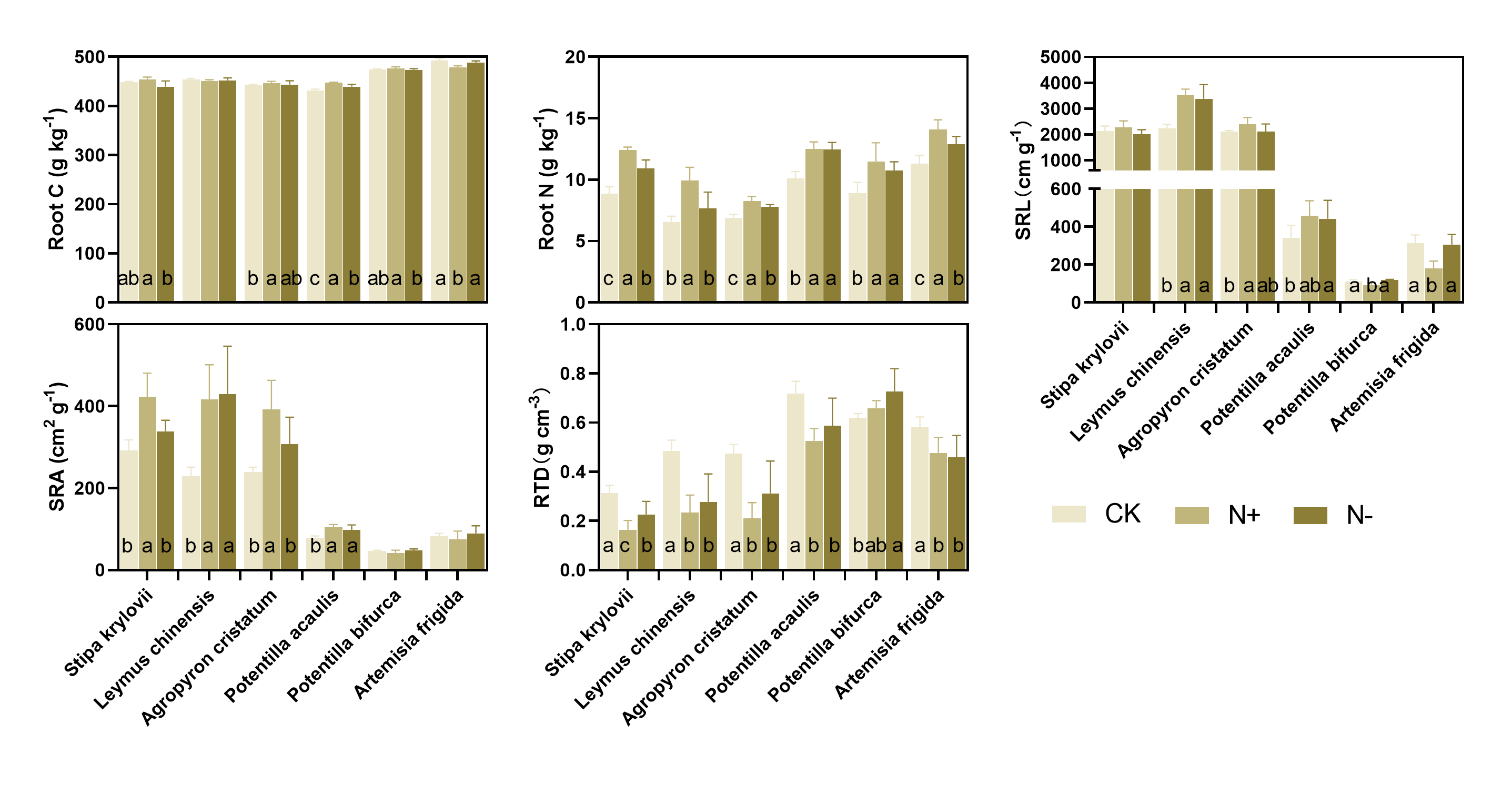


**Fig. S6.** Effects of N addition and N cessation on root variables of different plant species. Data are means ± SD for six replicates. CK, control; N+, N addition; N-, N cessation. Different letters indicate significant difference among treatments at *p* < 0.05. SRL, specific root length; SRA, specific root area; RTD, root tissue density.


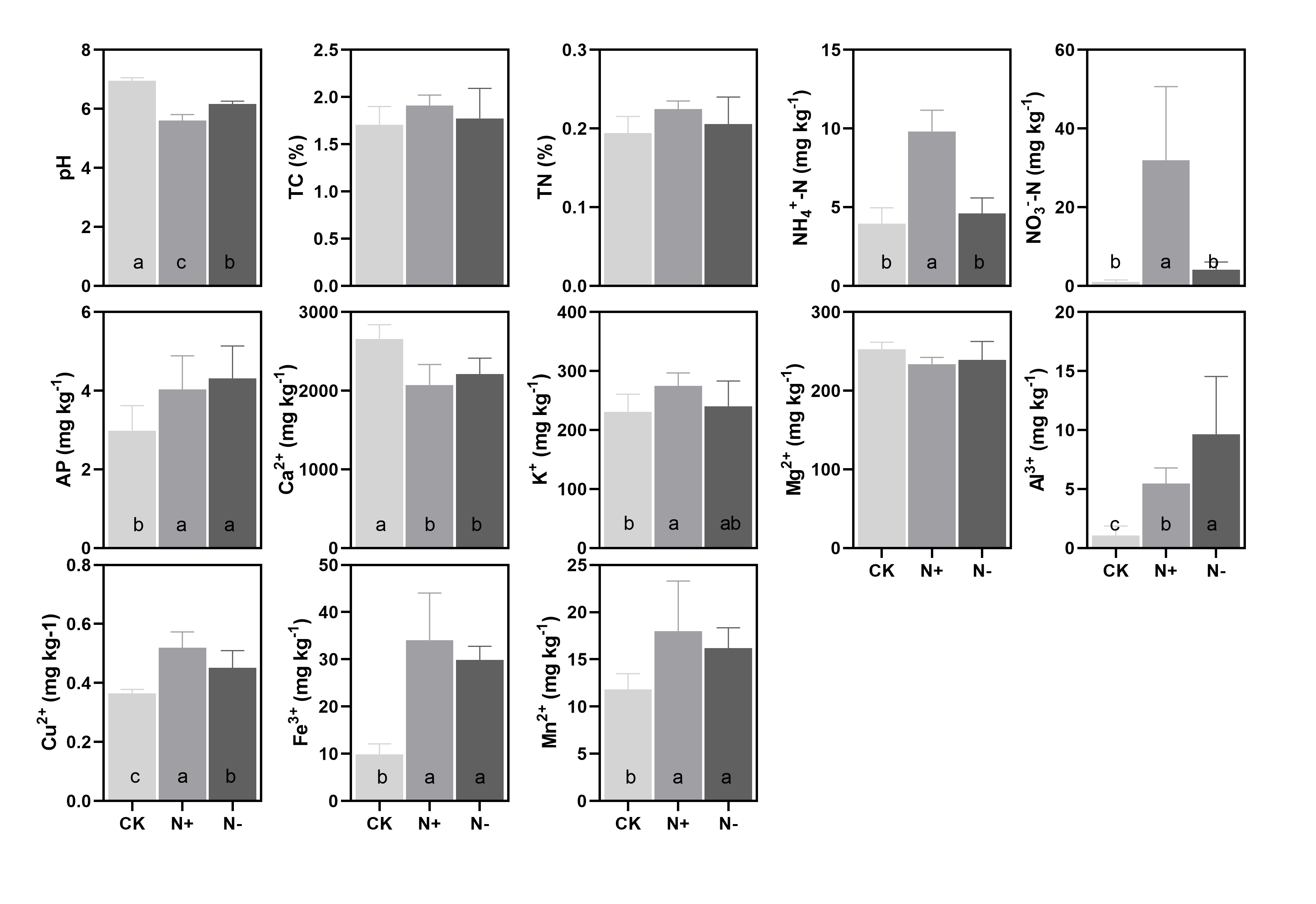


**Fig. S7.** Effects of N addition and N cessation on soil variables. Data are means ± SD for six replicates. CK, control; N+, N addition; N-, N cessation. Different letters indicate significant difference among treatments at *p* < 0.05.

**
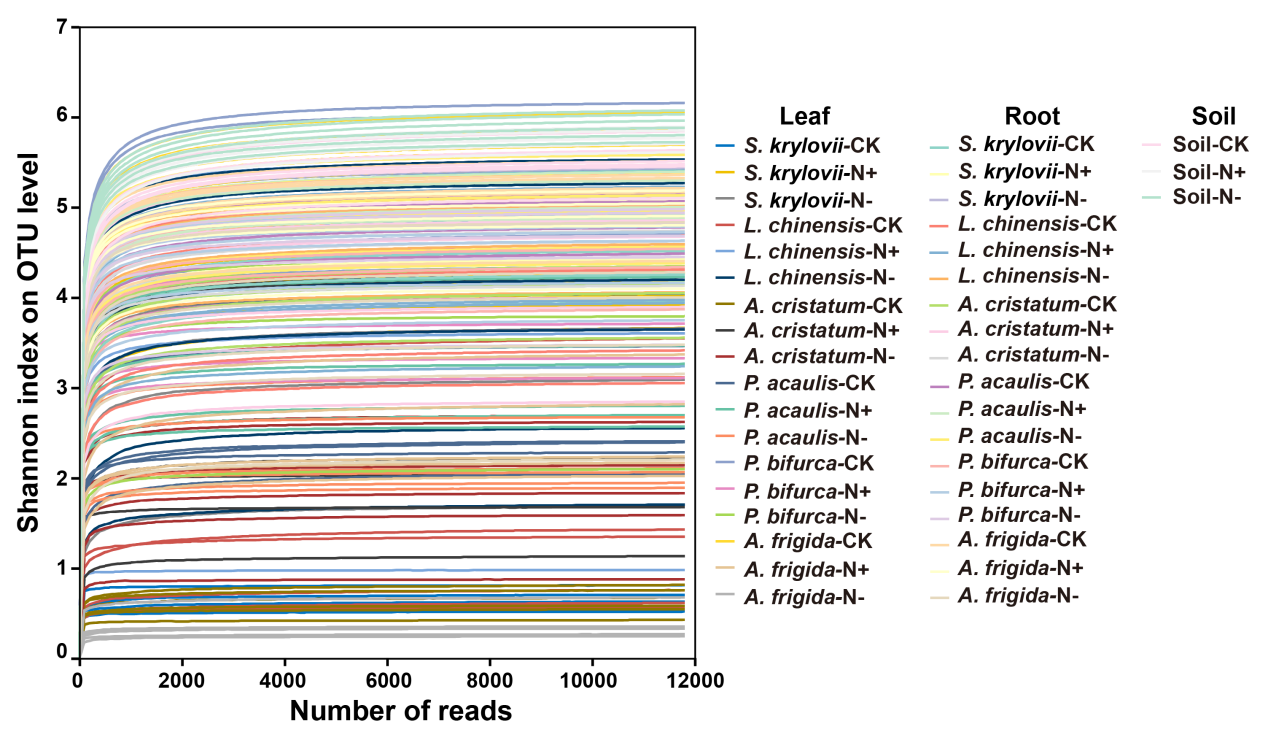
**

**Fig. S8** Rarefaction curves of all the samples showing sequence number and Shannon index**.** CK, control; N+, N addition; N-, N cessation.

**Table S1.** PERMANOVA for treatments on leaf- and root-associated bacterial communities of individual plant species based on Bray-Curtis semi-metric distance matrix.

|  | Species | CK *vs* N+ | | | CK *vs* N- | | | N+ *vs* N- | | |
| --- | --- | --- | --- | --- | --- | --- | --- | --- | --- | --- |
|  |  | F | R^2^ | *p* | F | R^2^ | *p* | F | R^2^ | *p* |
| Leaf | *S. krylovii* | 27.13 | 0.73 | 0.003 | 54.35 | 0.84 | 0.005 | 5.62 | 0.36 | 0.005 |
|  | *L. chinensis* | 13.54 | 0.58 | 0.007 | 14.13 | 0.59 | 0.003 | 2.62 | 0.21 | 0.01 |
|  | *A. cristatum* | 11.88 | 0.54 | 0.001 | 19.50 | 0.66 | 0.001 | 1.70 | 0.14 | 0.10 |
|  | *P. acaulis* | 6.10 | 0.38 | 0.006 | 10.89 | 0.52 | 0.005 | 2.34 | 0.19 | 0.05 |
|  | *P. bifurca* | 4.08 | 0.29 | 0.002 | 6.03 | 0.38 | 0.003 | 4.14 | 0.30 | 0.002 |
|  | *A. frigida* | 5.45 | 0.35 | 0.005 | 64.78 | 0.87 | 0.002 | 8.23 | 0.45 | 0.002 |
| Root | *S. krylovii* | 12.15 | 0.55 | 0.002 | 14.72 | 0.60 | 0.001 | 1.73 | 0.15 | 0.023 |
|  | *L. chinensis* | 7.78 | 0.44 | 0.004 | 5.75 | 0.36 | 0.003 | 2.21 | 0.18 | 0.008 |
|  | *A. cristatum* | 9.53 | 0.49 | 0.002 | 11.87 | 0.54 | 0.004 | 0.84 | 0.08 | 0.712 |
|  | *P. acaulis* | 2.80 | 0.22 | 0.003 | 1.97 | 0.16 | 0.029 | 1.17 | 0.10 | 0.279 |
|  | *P. bifurca* | 3.07 | 0.24 | 0.003 | 2.08 | 0.17 | 0.014 | 2.09 | 0.17 | 0.011 |
|  | *A. frigida* | 5.86 | 0.37 | 0.002 | 8.30 | 0.45 | 0.007 | 4.22 | 0.30 | 0.005 |

**Table S2.** PERMANOVA for plant identity on the leaf- and root-associated bacterial communities based on Bray-Curtis semi-metric distance matrix.

|  |  | CK vs N+ | | | CK vs N- | | | N+ vs N- | | |
| --- | --- | --- | --- | --- | --- | --- | --- | --- | --- | --- |
|  |  | F | R^2^ | *p* | F | R^2^ | *p* | F | R^2^ | *p* |
| Leaf | Species | 3.83 | 0.23 | 0.001 | 4.36 | 0.25 | 0.001 | 5.93 | 0.31 | 0.001 |
|  | Family | 6.22 | 0.15 | 0.001 | 6.71 | 0.16 | 0.001 | 7.60 | 0.18 | 0.001 |
|  | Plant group | 7.95 | 0.10 | 0.001 | 9.35 | 0.12 | 0.001 | 8.23 | 0.11 | 0.001 |
| Root | Species | 4.20 | 0.24 | 0.001 | 5.55 | 0.30 | 0.001 | 4.83 | 0.27 | 0.001 |
|  | Family | 7.65 | 0.18 | 0.001 | 9.87 | 0.22 | 0.001 | 7.55 | 0.18 | 0.001 |
|  | Plant group | 11.47 | 0.14 | 0.001 | 15.15 | 0.18 | 0.001 | 10.32 | 0.13 | 0.001 |

**Methods S1** Detailed information on the measurement of root traits and soil properties

**(1) Root trait measurement**

The roots from each species were soaked in deionized water for at least six hours to rehydrate until saturated. Then the water on the root surface was absorbed quickly using absorbent paper. Then these roots were scanned with a scanner (Epson Expres-sion 10000XL, Seiko Epson Corporation, Nagano-ken, Japan) in gray scale at a resolution of 600 dpi. Scanned images were analyzed by WinRHIZO (Regent Instruments Inc., Quebec City, QC, Canada) to calculate the length (L), volume (V) and surface area (SA). Thereafter the roots were dried at 65 °C to a constant weight and the dry weight (m, g) was mesured. Then the dried roots were grounded into fine powders with a ball mill (MM 400; Retsch, Haan, Germany). The specific root length, specific root area and root tissue density were calculated using the following equation, respectively.

Specific root length (SRL) = L/M;

Specific root area (SRA) = SA/M;

Root tissue density (RTD) = M/V.

The root total carbon and root total nitrogen contents were measured with an elemental autoanalyzer (Vario El III, Elementar, Hanau, Germany).

**(2) Determination of soil variables**

Soil pH was determined using a soil:water ratio of 1:2.5 (w/vol). Soil total carbon and soil total N were determined using an elemental analyzer (Vario EL Ⅲ, Elementar, Germany). The NH_4_^+^-N and NO_3_^-^-N concentrations in fresh soils were extracted by 2 M KCl at ratio of 1:5 (w/vol) and then measured with a continuous flow analyzer (AutoAnalyzer 3, SEAL Analytical GmbH, Norderstedt, Germany). To measure exchangeable metal ions (Fe^3+^, Mn^2+^, Al^3+^ and Cu^2+^), air-dried soil was incubated in an extracting solution (pH 7.3) consisting of 10 mM CaCl_2_, 0.1 M triethanolamine (TEA) and 5 mM diethylenetriamine pentaacetic acid (DTPA) with a soil:solution ratio of 1:2 (w/vol) for 2 hours. For determination of base cations including Ca^2+^, Mg^2+^ and K^+^, air-dried soil was incubated in 1 M NH4OAc (pH 7.0) solution with a soil:solution ratio of 1:10 (w/vol) for 30 minutes. After the extraction solution was filtered, the concentrations of Ca^2+^, Mg^2+^, K^+^, Fe^3+^, Mn^2+^, Al^3+^ and Cu^2+^ were measured by ICP-OES (Thermo Electron Corporation). Available phosphorus (AP) in soil was determined in 0.5 M NaHCO_3_ solution (pH 8.5).
